# Can Cannabidiol Affect the Efficacy of Chemotherapy and Epigenetic Treatments in Cancer?

**DOI:** 10.3390/biom11050766

**Published:** 2021-05-20

**Authors:** Courtney Griffiths, James Aikins, David Warshal, Olga Ostrovsky

**Affiliations:** 1 MD Anderson Cancer Center at Cooper, Division of Gynecologic Oncology, Cooper University Healthcare, Camden, NJ 08103, USA; griffiths-courtney@cooperhealth.edu (C.G.); aikins-james@cooperhealth.edu (J.A.); warshal-david@cooperhealth.edu (D.W.); 2 Department of Surgery, Division of Surgical Research, Cooper University Healthcare and Cooper Medical School, Rowan University, Camden, NJ 08103, USA

**Keywords:** cannabinoids, phytocannabinoids, endocannabinoid system, ovarian cancer, chemoresistance, targeted therapy, epigenetic therapy

## Abstract

The success of cannabinoids with chronic neuropathic pain and anxiety has been demonstrated in a multitude of studies. With the high availability of a non-intoxicating compound, cannabidiol (CBD), an over-the-counter medication, has generated heightened interest in its use in the field of oncology. This review focuses on the widespread therapeutic potential of CBD with regard to enhanced wound healing, lowered toxicity profiles of chemotherapeutics, and augmented antitumorigenic effects. The current literature is sparse with regard to determining the clinically relevant concentrations of CBD given the biphasic nature of the compound’s response. Therefore, there is an imminent need for further dose-finding studies in order to determine the optimal dose of CBD for both intermittent and regular users. We address the potential influence of regular or occasional CBD usage on therapeutic outcomes in ovarian cancer patients. Additionally, as the development of chemoresistance in ovarian cancer results in treatment failure, the potential for CBD to augment the efficacy of conventional chemotherapeutic and epigenetic drugs is a topic of significant importance. Our review is focused on the widespread therapeutic potential of CBD and whether or not a synergistic role exists in combination with epigenetic and classic chemotherapy medications.

## 1. Introduction

Ovarian cancer is responsible for the highest mortality rate among gynecologic cancers with an estimated 5-year survival rate of 48.6%. According to SEER data, the estimated new cases in 2020 will reach 21,750 with approximately 14,000 deaths [1]. Mortality from ovarian cancer is due to the advanced stage at diagnosis and high incidence of chemoresistant disease that eventually develops over time, prompting a pressing investigation for alternative therapies [2,3]. Standard of care therapy includes primary debulking surgery and platinum/taxane-based chemotherapy [4]. Despite optimal cytoreduction and systemic chemotherapy, approximately 70% of patients with advanced stage disease will recur in the first 3 years [4]. Novel targeted therapies such as immunotherapy and epigenetics have been topics of more recent discussion [5,6,7]. Given that combined therapies have a better impact on treatment outcomes, there has been a shift towards seeking alternative treatment modalities including cannabinoids given their demonstrated therapeutic benefit in the oncologic realm [8].

In the development of new cancer treatment strategies, the role of marijuana-associated cannabinoids draws special interest due to their wide therapeutic use for depression, nausea and vomiting, anorexia, and spasticity [9]. Promising effects in the realm of neurology have also been demonstrated such as in the alleviation of anxiety, trigeminal neuralgia, and psychiatric disorders [10]. Moreover, a growing number of patients are turning to these exogenous cannabinoids as an alternative regimen for pain relief, given their more widespread availability and relatively benign safety profile compared to opioids [11]. Among 244 medical cannabis users, a 64% decrease in opioid use was documented as well as 45% of participants reporting an improved quality of life [12]. Despite the paucity of the studies, this trend is likely to continue in the setting of a worsening opioid crisis (given that approximately 69.5% of drug overdose deaths were due to opioids in 2018 alone), suggesting that clinicians are seeking alternative sources of analgesia, especially with regard to cancer-related pain [13,14].

Of the utmost importance is understanding what interactions cannabinoids may have with traditional chemotherapeutic agents and other targeted therapies. While antitumorigenic effects have been reported in the oncology literature, these data have somewhat limited utility in humans with regards to fully comprehending clinically relevant plasma versus tissue concentrations, as previous work has focused on artificial or non-physiologically relevant concentrations.

As there is also insufficient data evaluating the synergistic effects of cannabinoids with cancer treatments, we aim to review the immunomodularity properties of cannabidiol (CBD) and whether or not its use in conjunction with immune or chemotherapy has a synergistic effect on drug cytotoxicity. In addition, we hope to gain insight into whether or not the use of CBD with epigenetic therapy could enhance response rates at clinically relevant dosages.

### 1.1. Cannabinoids and Their Working Mechanisms

The endocannabinoid system (ECS) has been a topic of interest for oncologists given its success in the treatment of chronic pain and its use as an antiemetic for chemotherapy-induced nausea or vomiting [15]. In 2017, the National Academies of Sciences, Engineering and Medicine released a comprehensive review of the health effects of recreational and therapeutic cannabis use, highlighting the benefit in the treatment for chemotherapy-induced nausea and vomiting (CINV) and chronic pain [15]. Recent attention has been paid to the antitumor properties of cannabinoids and whether their use may enhance more specialized and targeted therapies for those who develop resistance to standard of care treatments.

The endocannabinoid system is a widespread neuromodulatory system which is comprised of unsaturated fatty acid derivatives with a wide distribution in the human body. Endogenous cannabinoids, cannabinoid receptors (see Table 1), and the enzymes responsible for synthesis and degradation make up the ECS system [16,17]. There are three main chemical classes of cannabinoids that act on cannabinoid receptors: (1) exogenous compounds, also referred to as phytocannabinoids (extracted from the *Cannabis sativa L*. plant), including 9-tetrahydrocannabinol (THC), cannabidiol (CBD), and cannabinol (CBN); (2) naturally produced endocannabinoids; and (3) synthetic cannabinoids produced under laboratory conditions. The two endogenous endocannabinoids which have been most widely studied are *N*-arachidonoylethanolamine (AEA) and 2-arachidonoylglycerol (2-AG). They bind to and activate both cannabinoid receptors-1 and 2 (CB1, CB2), although AEA acts only as a partial agonist at CB1 and weakly at CB2 while 2-AG acts equally at both. Therefore, 2-AG is deemed to be the primary endogenous agonist of both receptors. The receptor actions of both AEA and 2-AG are mirrored by THC, all lipophilic compounds with the endogenous counterparts becoming rapidly inactivated, suggesting a more sustained effect [17].

These particular cannabinoid receptors are G-protein coupled receptors which influence incoming signals. The CB1 receptor in particular is mainly populated in the central nervous system while the CB2 receptors are largely associated with maintaining homeostasis within the immune system [18]. Of interest, CB1 receptors are densely present in reproductive organs such as the ovaries, endometrium, and testes. As cancerous cells are thought to express greater levels of CB1 and CB2 in comparison to normal cells, this may prove to be a useful target for impairing tumor propagation [19].

Aside from the cannabinoid receptors, CBD (see Table 1) has demonstrated agonist activity at the 5-HT1a receptor in a concentration-dependent manner. Various doses of CBD were used to compare motor effects against a particular serotonin agonist with high doses (>10 mg/kg) influencing vertical motor activity. This particular effect was deemed to be a possible mechanism of anxiolytic, anti-emetic, and antidepressant activity [20,21]. Furthermore, chemotherapy-induced neuropathic pain has been another focus of CBD usage among cancer patients. One author identified a suppression in Paclitaxel-induced allodynia with the use of two separate CB2 receptor agonists, indicating a potential therapeutic target for grade 2 or 3 neuropathies [22]. Not only do studies demonstrate a synergistic effect of cannabinoids with other analgesic medications, but they have proven successful in antagonizing the negative side effects of opioids, pertinent for mitigating a worsening national opioid crisis [23]. Lastly, a partial agonist effect has been demonstrated on dopamine receptors, allowing for a similar antipsychotic effect as certain antipsychotic medications such as Aripiprazole in a biphasic manner [24]. One particular study identified a more significant clinical improvement in schizophrenic symptoms with the addition of CBD in comparison to a potent antipsychotic medication with a more favorable side effect profile [25].

### 1.2. Are CBD and Marijuana Usage the Same?

Multiple studies demonstrate that two main cannabinoid components in marijuana, non-intoxicating CBD and intoxicating THC, play a role in anti-inflammatory and immunomodulatory processes [26,27,28]. CBD has been extensively studied in pre-clinical animal models [29,30], and its salutary effect on wound healing and regenerative medicine has been demonstrated [31,32]. In addition, the product is now highly available in the US market for a multitude of conditions including but not limited to depression, chronic pain, and anxiety [33]. Moreover, this beneficial role of CBD may be particularly relevant from a surgical perspective in oncology patients, where an individual’s wound healing ability or neuropathic pain is caused or often affected by chemotherapeutic agents [34].

**Figure 1:**
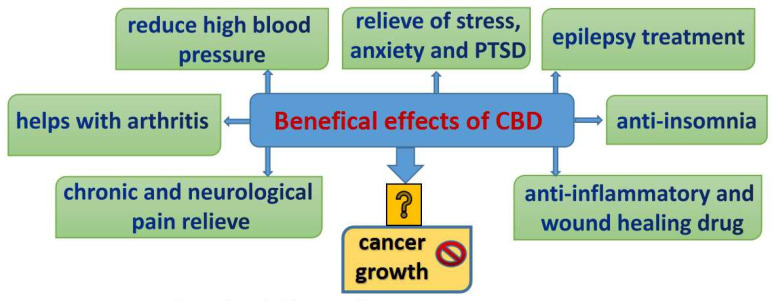
Major beneficial effects of CBD usage [37,38,39,40].

Phytocannabinoids are known to play a role in many physiologic conditions, mainly contributing to appetite stimulation, analgesia, and chemotherapy-induced nausea and vomiting in oncology patients. While THC is known to have an intoxicating effect, CBD does not exhibit the same effects and is adept at antagonizing the very psychomimetic effects that THC causes [35]. Aside from this, CBD has demonstrated anti-inflammatory, anticonvulsant, antioxidant, antiemetic, and anxiolytic properties, prompting widespread interest particularly in cancer care with an additional focus on the antitumorigenic properties [36] (Figure 1).

Despite the aforementioned wide-ranging benefits in cancer patients, the therapeutic effects or possible outcomes of combination usage of marijuana or marijuana-based compounds such as CBD with cancer therapies has not been extensively investigated [41]. It is paramount to understand how cancer patients’ usage of various CBD-containing products might impact the efficacy of cancer treatment, while reducing toxicities such as neuropathy [42].

Given that marijuana as well as pure CBD products contain varying concentrations of CBD regardless of recreational or medicinal use, it is not only important to determine the relevant dosage or concentration, but also the frequency of usage (intermittent versus regular users) which may affect patients who are undergoing chemotherapy, epigenetic, and immunotherapies. Specifically, the lipophilicity of the compound may contribute to greater accumulation in fat, leading to a very different impact on regular versus intermittent users, not to mention among patients of varying weight [43]. With the rising prevalence of CBD use in the United States and its minimal adverse side effects in comparison to THC, we will focus on the former for purposes of this review. 

### 1.3. Antitumorigenic and Biphasic Properties

Many studies have demonstrated the ability of cannabinoids to affect the rate of cell proliferation, migration, angiogenesis, and apoptosis, particularly in breast, prostate, and glioma cell lines [44,45,46,47]. Initial antitumor effects were reported by Munson et al. who demonstrated an inhibition of lung adenocarcinoma cell growth in vitro by THC and confirmed in vivo using the Lewis lung carcinoma murine model [48]. The mechanism of action has been shown to be through the modulation of different proteins involved in the ECS such as CB1, CB2, GRP55, the ionotropic receptor TRPV1, or the fatty acid amide hydrolase (FAAH). Many studies suggest that CB receptors and their ligands are upregulated in tumor tissue, and that overexpression can lead to more aggressive tumors [49]. However, alternatively, there have been tumor-suppressive roles identified likely secondary to the upregulation of endocannabinoid-degrading enzymes [50]. Although more has been studied with THC, CBD and THC may have varying effects on cancer progression due to their activation of different receptors [51]. THC binds directly to the CB1 receptor while CBD does not. However, CBD interacts allosterically with CBR1 and changes the shape of the receptor in a way that weakens the ability of THC to bind with CBR1 [52]. In addition, CBD inhibits the degradation of endogenous AEA, further enhancing its positive effect on neuroprotective and anti-inflammatory properties [53].

Cannabinoids have been shown to produce a biphasic effect depending on the concentration of the compound [54,55]. Given that most phytocannabinoids are not CB1 or CB2-selective agonists, outcomes are mainly based on receptor availability and on the overall state of the cell [56]. Specifically, high concentrations (micro-molar) of endogenous cannabinoids have been found to display inhibitory effects on tumor growth while low concentrations (nano-molar) induce growth [57]. Based on the similar properties of CBD to anandamide, this too likely exhibits biphasic effects at different concentrations, with a particular effect on cancer at physiologic concentrations. There appears to be a delicate balance between protumorigenic and antitumor effects of cannabinoids in preclinical studies, highlighting the clinical importance of correlating ideal and relevant CBD concentrations for daily usage as well as for both intermittent and regular users [58]. For example, if 10 µM works to suppress cancer growth in breast cancer, does the over-the-counter oral drop formulation with clinically relevant concentrations such as 15–50 mg lead to the same effect? To date, the effect of cannabinoids on cancer cell propagation as well as their ability to mitigate chemotherapy effects at clinically relevant concentrations have not yet been elucidated.

Despite the prevalence of CBD use, dose recommendations are mostly lacking due to the paucity of studies, therefore both the therapeutic concentration and route of administration in order to achieve minimum effective dose has not been established [59]. In a comprehensive review comparing clinical outcomes of CBD, a positive effect was noted in 66% of studies with doses ranging between <1 and 50 mg/kd/d, suggesting that CBD has a wide therapeutic range [59]. However, this wide monotherapeutic range may not only have varying effects on different disease processes, but may lead to vastly different outcomes when combined with other cancer therapies. None of the studies, to our knowledge, were focused on the effects on tumor growth and migration.

Importantly, these antitumorigenic effects have been demonstrated at artificially high concentrations of CBD. McAllister et al. demonstrated a dose-dependent effect of CBD on a triple negative breast cancer cell line with higher concentrations (1.5 µmol/L as opposed to 0.1 or 1.0 µmol/L) causing a significant decrease in breast cancer proliferation and invasion [60]. Another study evaluating the effect of CBD on the tumor microenvironment revealed that lower concentrations of CBD (3, 6 µM) had less of a direct effect on proliferation in comparison to higher concentrations (12, 15 µM) [61]. On the contrary, the clinically relevant plasma concentration of CBD after daily digestion of 700 mg yielded mean plasma levels of 6–11 ng/mL, which is about 19–35 nM [43] and roughly 500–1000 fold lower than reported tested concentrations. Therefore, due to its biphasic nature, the compound can be inherently dangerous without full analysis of the effects of clinically relevant concentrations obtained in patients’ blood as well as their overlap with standard of care oncology treatments with regards to outcomes. Biphasic effects have been identified in many processes including motor activity, motivational processes, and anxiety responses [62]. The authors of one study identified an anxiogenic-like effect on mice behavior after the administration of high doses of cannabinoids, whereas an anxiolytic effect was noted with low administered doses [63]. While CBD has a relatively good safety profile, adverse effects have been documented such as lethargy, diarrhea, somnolence, and drug–drug interactions, hence the need for further dose-dependent clarification [63]. Thus far, to our knowledge, there is very limited understanding of how clinically relevant CBD usage from commercially available CBD-based products may influence the development and propagation of cancer cells. Therefore, more studies reflecting clinical concentrations after the ingestion and inhalation of widely available CBD-based distributed products are encouraged as the findings may influence cancer treatment development. As CBD is a lipophilic compound, it is not surprising that human data have shown plasma levels of CBD to be increased when administered in a fed state [64]. Animal studies have confirmed these findings with a 3-fold increase in bioavailability through an oral route [65]. There is a paucity of data regarding other methods of administration in humans, although animal studies have demonstrated a benefit of transdermal and topical CBD formularies; therefore, further analysis is warranted [66]. Moreover, given the variation in results with different artificially high or non-physiologically relevant concentrations of CBD, we have yet to determine whether CBD maintains antitumorigenic potential at clinically relevant doses seen in over the counter products.

## 2. Role of CBD in Cancer Treatment and Its Potential Influence on Cancers’ Therapies Outcomes 

### 2.1. CBD Success in Cancers

The majority of literature published evaluating the antiproliferative effects of CBD on cancer pertains mainly to breast, colorectal, and brain neoplasms. Limited data are available on the use of cannabinoids in ovarian cancer, however one study identified a strong expression of cannabinoid receptor type I in epithelial ovarian tumors while benign and borderline ovarian neoplasms only demonstrated a moderate expression. The author also noted a correlation between overexpression and level of tumor invasion, highlighting the involvement of the endocannabinoid system in cancer propagation [19]. A case report demonstrated the response of a woman with low grade serous ovarian cancer who declined conventional chemotherapy where a significant positive response was noted with the use of Laetrile and CBD oil in the metastatic setting [67]. A very recent publication evaluated the antitumor effect of CBD monotherapy and CBD in combination with conventional chemotherapy in ovarian cancer utilizing microparticles as CBD carriers. Results favored a 2-fold decrease in tumor growth with the use of CBD in combination with paclitaxel [68]. This was the first study, to our knowledge, which specifically evaluated the effects of CBD on traditional chemotherapy in ovarian cancer, supporting a synergistic relationship with a positive effect on treatment efficacy.

The antiproliferative effects of cannabinoids on cancer have been demonstrated in many studies. Both in vitro and animal studies have reported a variety of mechanisms including apoptosis, angiogenesis, and influence on metastatic potential [41]. Specifically, the work of Romano et al. revealed reduced cell proliferation in colorectal cancer cells with the use of CBD in mice models [69]. Other models have hypothesized various mechanisms of action against colorectal cancer cells, however, they include direct activation of CB1 and CB2 receptors [70]. Chemopreventive effects were effectively demonstrated in experimental colon cancer models indicating a potential use for CBD in cancer prevention rather than treatment [71]. Another study exhibited significant antitumor effects of CBD on human glioma cells in both in vitro and in vivo models. This antitumorigenic effect was found to be triggered by the induction of apoptosis [72]. Alternative delivery systems were identified in a study highlighting the use of THC and CBD-enhanced microparticles which led to apoptotic, antiproliferative, and antiangiogenic effects on glioma cell tumors [73]. Marcu et al. demonstrated that 0.4 µM CBD inhibits the growth of several gliobastoma cell lines and that CBD was a much more potent inhibitor of tumor cell growth than THC [74]. Even further, breast cancer studies have demonstrated promising results with regards to cancer cell proliferation and invasion through differential modulation of the extracellular signal-regulated kinase (ERK) and reactive oxygen species (ROS) pathways. In addition, these pathways subsequently lead to the down-regulation of Id-1 expression, which is a key regulator in the metastatic potential of breast cancers [75].

The endocannabinoid system and its effect on gynecologic cancers is not yet widely understood; however, there has been evidence of its influence on both cervical and endometrial malignancies [19,76,77,78]. While the response rates vary greatly depending on the malignancy, each cancer type has its challenges with regard to treatment options after failing traditional therapy. Similarly to ovarian cancer, patients who receive a diagnosis of advanced stage cervical cancer tend to have limited treatment options after failing traditional chemo and radiation therapy. Specifically, Contassot et al. demonstrated a strong expression pattern of both CB1 and CB2, as well as TRPV1 in cervical cancer cell lines. Further, AEA was shown to have a pro-apoptotic effect via the expression of vanilloid receptor-1 (VR1), whereas its binding to CB1 and CB2 had a protective effect [77].

Despite the majority of endometrial cancers being diagnosed at an early stage with a 5-year survival of 95%, recurrent or advanced endometrial cancer is rarely curable [79]. Fonseca et al. studied the effect of cannabinoids on two endometrial cancer cell lines representing Type I (estrogen dependent) and Type II (non-estrogen driven) cancers, as well as a non-cancerous line as a control. The cells were treated with four different cannabinoids, and a decrease in cell viability was noted with the use of CBD and endocannabinoids at concentrations higher than 5 µm/L, while THC had no effect even at the highest concentrations. Further, the author found evidence of apoptosis only in Type I cells with no programmed cell death activity in Type II cancer cells, indicating a possible relationship with estrogen [78]. An interesting relationship between TRPV2 (a known CBD ligand) and Type II endometrial cancers, specifically serous and other high grade subtypes, was noted in one particular study which could indicate a new potential marker and therefore benefit for the use of CBD as an adjunct to standard chemotherapeutic drugs. Specifically, in vitro TRPV2 over-expression showed an increased migratory ability and response to Cisplatin with improvement in cytotoxic effects with CBD [80].

Agreeably, tumor pathogenesis varies among different malignancies, and while there has been very promising data regarding the use of CBD in breast, brain, and colorectal cancers, there is still much that remains to be seen in the realm of gynecologic cancers, especially at the forefront of aggressive ovarian tumors.

### 2.2. Does CBD Usage Affect the Potency of Classic Chemotherapy?

While antitumorigenic effects on cancer cells have been demonstrated with the use of CBD monotherapy, it is unclear how this compound can affect traditional cancer treatments when used in conjunction, most importantly for regular users. As with many medications and compounds, CBD is a known inhibitor of the cytochrome P450 (CYP) system [81]. This poses the question of how cannabinoids may affect classic chemotherapy given that this inhibition could potentially lead to increased plasma concentrations of chemotherapeutic agents and therefore increased toxicities. Studies have reported the synergistic effects of cannabinoids and cytotoxic drugs [82]. As demonstrated in the paper by Fraguas-Sanchez et al., the addition of CBD to conventional chemotherapy with Paclitaxel only enhanced the antiproliferative effects of ovarian cancer cells and did not by any means, hinder the cytotoxic effect of chemotherapy [68]. Further, breast oncology literature proved that CBD did not attenuate the efficacy of Paclitaxel in the inhibition of breast cancer cell viability [42]. Similar outcomes have been seen with gliobastoma cells which exhibited an increased sensitivity to chemotherapeutic agents Carmustine, Temozolomide, Doxorubicin, and Cisplatin with the use of CBD [82,83]. Specifically, a two-part randomized, double-blind, placebo-controlled study of 1:1 CBD/THC plus Temozolomide was performed in patients with recurrent gliobastoma multiforme. Median survival was notably improved with the CBD/THC group as compared to placebo (550 vs. 369 days) with no grade 3 or 4 side effects [84]. Cannabinoids have also helped reduce the burden of chemotherapy-associated side effects, specifically exhibiting a protective effect against Paclitaxel-induced neurotoxicity [85]. Although it is a different tangent, regarding the effects on radiation therapy, cannabinoids were shown to increase the radiosensitivity of glioma cells and in turn, large reductions in tumor volumes when used in conjunction with radiation treatment [86]. Even more pertinent to the pursuit of alternative therapies in the setting of chemoresistant disease, CBD has been found to decrease viability in paclitaxel-resistant breast cancer cells in a concentration-dependent manner through the induction of apoptosis, emphasizing the need for further study in resistant cancer cell lines [36]. While no synergistic effect was identified between CBD and Paclitaxel, cannabinoids were still able to induce apoptosis in platinum-resistant cells when Paclitaxel was unsuccessful.

Additional preclinical trials are needed to confirm the optimal concentration of CBD in both regular and intermittent users and whether or not dose-dependent concentrations alter the efficacy of standard chemotherapeutic agents.

### 2.3. CBD and Targeted Therapies

Of particular importance is the common notion of chemoresistance to standard therapy. The majority of ovarian cancer patients who develop resistance to traditional chemotherapy will unfortunately succumb to their disease; therefore, much attention has been redirected towards reversing this process. Many in vitro and in vivo models have been used to evaluate the effects of CBD on T-cells and macrophages with results indicating an innate ability to alter immune system reactivity [87]. Studies have demonstrated a focal decrease in tumor necrosis factor α (TNF-α) and interferon gamma (IFN-γ) production with the use of CBD, among reduction in IL-1 and TNF-α in human peripheral blood mononuclear cells, which are inherently known to augment anti-apoptotic molecules in cancer cells [88,89]. The immunomodulatory potential of CBD begs the question of whether or not its use in conjunction with immunotherapeutic drugs can only further improve patient centered outcomes.

### 2.4. Future of Epigenetic Treatments on Ovarian Cancer

Epigenetic therapy (ET) is a topic of great potential with regard to the field of oncology, especially among chemoresistant disease [7]. Human cancer cells harbor global epigenetic abnormalities as well as numerous genetic alterations. These alterations interact at all stages of cancer development, working coherently in order to promote cancer progression [90,91]. Epimutations can lead to the silencing of tumor suppressor genes independently and can also promote tumorigenesis by activating oncogenes [90]. Aberrant epigenetic processes or failure to maintain heritable epigenetic marks can lead to altered gene function and malignant cellular transformation [92]. Epigenetic therapy can essentially reprogram DNA methylation, histone modifications, nucleosome positioning, and many other processes, in turn substantiating superb benefits in reversing epigenetic aberrations in many cancers [93,94]. Therefore, these treatments may have the potential to not only suppress cancer propagation and metastasis, but also to reverse chemoresistance and perhaps allow patients to once again be susceptible to traditional platinum-based therapy.

On the other hand, as discussed in a review on cannabis teratology, increasing levels of cannabis usage were associated with an increase in teratogenic effects. The authors touch on the effect of THC and other addictive agents on microtubule interference leading to genotoxicity and epimutations; however, the relation of CBD itself is not explicitly discussed calling into play whether a true detrimental epigenetic effect does indeed exist [95].

To our knowledge, there have been no studies to date evaluating the combined effect of CBD and ET on cancer cells. Further drug interaction studies are warranted to investigate whether or not a symbiotic relationship exists between CBD and epigenetic drugs, enhancing the antiproliferative effect on ovarian cancer cells.

## 3. Conclusions

CBD may hold significant promise with regards to both monotherapy and combined treatment when used in conjunction with standard chemotherapeutics as well as with epigenetic and immunotherapy. While a solo treatment benefit may not be the penultimate goal, using CBD as an adjunct may further augment the efficacy of standard therapies, aid in the reversal of chemoresistant disease, promote wound healing after surgery, and help alleviate toxic side effects simultaneously, resulting in an overall improved quality of life for oncology patients. Given the notable benefit previously established with regard to antiemetic, anxiolytic, and analgesic properties, understanding the potential role of CBD as an immunomodulator to help balance the immune response in order to fight cancer is an area of significant interest, especially for patients who have failed traditional lines of therapy. Further studies are warranted to help determine at what safe concentration and administration route CBD will have a clinically relevant effect without negatively impacting the cytotoxic effects of other targeted therapies.

## Figures and Tables

**Table 1:** Major receptor targets of cannabidiol (CBD).

Receptor	Receptor Activity
CB1	Negative allo modulator
CB2	Weak antagonist
FAAH	Weak inhibitor
FABP	Inhibitor
5-HT1A	Full agonist
5-HT2A	Weak partial agonist
5-HT3A	Negative allo modulator
D2High (Dopamine)	Partial agonist
uOR (Opioid)	Negative allo modulator
δOR (Opioid)	Negative allo modulator
GPR55	Negative allo modulator
PPAR-gamma	Full agonist
Adenosine A2A receptors	Negative allo modulator
TRPV1	Negative allo modulator
TRPV2	Full agonist

## Data Availability

Not applicable.
